# Associated Factors and Quality of Life in Self-Reported Psoriasis Among Adults in Saudi Arabia: An Exploratory Cross-Sectional Study

**DOI:** 10.3390/jcm15197622

**Published:** 2026-10-01

**Authors:** Rena H. Alharbi, Mohammad A. Jareebi, Khlood K. Alattas, Shatha H. Moharaq, Alia A. Alruwished, Ghufran A. Almowalad, Sarah H. Bahakeem, Amal A. Badri, Saja A. Almraysi, Rauf M. Mawkili, Nuha H. Abutalib, Amany Mashi, Farjah H. Algahtani, Abdulwahab A. Aqeeli, Alaa H. Hakami

**Affiliations:** 1 Faculty of Medicine, Jazan University, Jazan 45142, Saudi Arabia; rena.hr000@gmail.com (R.H.A.); khloodalattas40@gmail.com (K.K.A.); shathamoharaq@gmail.com (S.H.M.); alia.sut2004@gmail.com (A.A.A.); dr.ghufran2002@gmail.com (G.A.A.); bahakeemsarah@gmail.com (S.H.B.); amal2mm2@gmail.com (A.A.B.); sajaalasiri1@gmail.com (S.A.A.); raoof1438@gmail.com (R.M.M.); 2 Department of Family and Community Medicine, College of Medicine, Jazan University, Jazan 45142, Saudi Arabia; nabutaleb@jazanu.edu.sa (N.H.A.); aaqeeli@jazanu.edu.sa (A.A.A.); 3 Department of Dermatology, Armed Forces Hospital, Jazan 45142, Saudi Arabia; dr.a.almashi92@hotmail.com; 4 Oncology Center, Epidemiology and Public Health Research, Faculty of Medicine, King Saud University/King Saud Medical City, Riyadh 12373, Saudi Arabia; falgahtani@ksu.edu.sa; 5 Department of Surgery, College of Medicine, Jazan University, Jazan 45142, Saudi Arabia; alaahakami@jazanu.edu.sa

**Keywords:** psoriasis, prevalence, quality of life, WHOQOL-BREF, smoking, associated factors, Saudi Arabia, cross-sectional study

## Abstract

**Background and Objectives**: Psoriasis is a chronic immune-mediated inflammatory disease whose burden extends beyond the skin. Community-based data from Saudi Arabia are scarce, as most local studies recruit from dermatology clinics and miss people who never reach specialist care. This study examined its frequency in a multi-regional sample of adults in Saudi Arabia, described its clinical profile, identified factors associated with it, and examined its association with quality of life. **Methods**: An exploratory cross-sectional study recruited 1619 adults residing in Saudi Arabia by online convenience sampling, using a self-administered online questionnaire covering sociodemographic and health characteristics, psoriasis status and clinical features, lifestyle factors, and the Arabic WHOQOL-BREF. Groups were compared using the chi-square, Fisher’s exact and Mann–Whitney U tests. Multivariable logistic regression, with Firth-penalized and parsimonious sensitivity models, examined factors associated with psoriasis, and five linear models were fitted for the overall quality of life facet and each domain. **Results**: Psoriasis was reported by 3.3% of participants (95% CI 2.5 to 4.3), and by 2.7% (95% CI 2.0 to 3.6) when restricted to physician-diagnosed cases. Onset occurred before age 35 in 84.6% of cases, psychological stress was the commonest trigger (69.2%), and herbal remedies had been used more often (34.6%) than oral systemic agents (25.0%), phototherapy (15.4%) or biologics (7.7%). Current smoking was associated with higher odds of reporting psoriasis (aOR 4.23, 95% CI 1.97 to 9.08, *p* < 0.001) and male sex with lower odds (aOR 0.41, 95% CI 0.19 to 0.90), and this was consistent across sensitivity models; middle income and university education were also associated with higher odds, although these may reflect differences in who receives a diagnosis rather than differences in risk. Psoriasis was independently associated with a lower overall quality of life and general health score (β = −6.73, 95% CI −12.75 to −0.71, *p* = 0.028); all four domain coefficients were negative but non-significant. **Conclusions**: Psoriasis was reported by roughly one in thirty participants and was associated with poorer self-rated quality of life. Smoking was the most consistent modifiable correlate, and treatment patterns suggest reliance on remedies outside established care.

## 1. Introduction

Psoriasis is a chronic, relapsing, immune-mediated inflammatory disease characterised by well-demarcated erythematous plaques covered by silvery scale. Lesions favour the elbows, knees, scalp and lower back, although any skin surface may be affected [1]. The disease arises from a complex interaction between innate and adaptive immunity, with keratinocyte hyperproliferation driven by the interleukin-23 and interleukin-17 axis [1,2]. Global prevalence estimates vary widely by region, ranging from below 0.5% in parts of East Asia to above 3% in northern Europe, and the disease affects an estimated 60 million people worldwide [3]. In 2014 the World Health Organization formally recognised psoriasis as a serious non-communicable disease and called for better data on its burden [4]. Regional estimates for the Middle East remain comparatively sparse, and the Global Burden of Disease analyses have identified the Arabian Peninsula as an area of considerable uncertainty [5].

The onset and course of psoriasis are shaped by modifiable factors and genetic susceptibility. Smoking, obesity, psychological stress, infection and certain medications have all been implicated in disease development and exacerbation [6]. Smoking has the most consistent evidence base: a systematic review and meta-analysis found that current smokers carry substantially higher odds of psoriasis than never-smokers, and that smoking also contributes to incident disease [7]. These exposures are directly relevant to Saudi Arabia, where tobacco use, sedentary behaviour and metabolic disease have all risen alongside rapid urbanisation. Understanding how such factors distribute among affected individuals is a prerequisite for targeted prevention.

Beyond the skin, psoriasis imposes a burden comparable to that of major chronic diseases. Visible lesions, itch, unpredictable flares and the demands of long-term treatment affect self-image, social participation and daily functioning [8]. Anxiety and depressive symptoms are common, and disease severity alone explains only part of the variation in patient-reported outcomes [9]. Within Saudi Arabia, the available evidence comes largely from clinic-based samples in single regions. A study in the north described the clinical features of psoriasis and its effects on quality of life among patients attending dermatology services [10], while a separate cross-sectional study documented comorbidity patterns in a clinical cohort [11]. Such designs cannot estimate how common the disease is in the community, and they exclude individuals who never reach specialist care. Population-based data combining prevalence, clinical profile and a validated generic quality of life instrument are lacking.

Quality of life is now regarded as a core outcome in psoriasis because clinical severity scores capture only part of what patients experience. Early work quantifying this burden found reductions in physical and mental functioning of a magnitude seen in other major medical diseases [12], and in a survey of 6497 Nordic patients, self-reported rather than clinician-rated severity was the strongest predictor of impaired quality of life [13]. Psychological morbidity accounts for much of this burden. Around one in four patients report depressive symptoms and about one in eight meet criteria for clinical depression [14], and anxiety and other psychiatric comorbidities are also common [15]. Stress operates in both directions, arising from life with a visible chronic condition and acting as a reported trigger of flares, which can sustain a cycle of worsening skin and worsening wellbeing [16]. Quality of life in psoriasis is measured either with dermatology-specific instruments such as the Dermatology Life Quality Index [17], which are sensitive to skin-related impairment, or with generic instruments such as the WHOQOL-BREF [18], which allow comparison with the general population and with other chronic conditions. Generic instruments have rarely been applied to psoriasis in community samples from the Arabian Peninsula.

This study therefore aimed to estimate the prevalence of psoriasis among adults aged 18 years and above residing in Saudi Arabia, to describe the clinical characteristics, triggers and treatment patterns of affected individuals, to identify sociodemographic and lifestyle factors associated with the disease, and to quantify its association with quality of life using the Arabic WHOQOL-BREF. By recruiting from the general population rather than from clinics, the study addresses a gap that limits the interpretation of existing local evidence and provides estimates that can inform preventive and dermatological service planning.

## 2. Materials and Methods

### 2.1. Study Design and Setting

This exploratory analytical cross-sectional study was conducted among the adult population of Saudi Arabia. Data were collected in February 2026 using a self-administered online questionnaire. Recruitment was carried out by trained data collectors and distributed through widely used social media platforms, including WhatsApp, X, Snapchat, Instagram, Telegram and Facebook. Recruitment was initiated from Jazan in the southern region and propagated outward, so respondents from the southern region are over-represented relative to the national population distribution. Responses were nonetheless obtained from all five administrative regions of the Kingdom.

### 2.2. Study Population and Sampling

A non-probability convenience sampling technique was used. Eligible participants were men and women aged 18 years or above who resided in Saudi Arabia, whether Saudi nationals or legal residents, who were able to read Arabic and who provided informed consent. No restriction was placed on dermatological status, since the objective was to estimate prevalence in the general population rather than to recruit a clinical case series.

The minimum sample size was calculated using the standard formula for a single population proportion, n = Z^2^ × p(1 − p)/E^2^, with Z = 1.96 for a 95% confidence level, an expected prevalence of 0.50 and a margin of error of 0.05. This yielded a minimum of 385 participants. To improve precision for a relatively uncommon outcome and to allow for incomplete responses, recruitment continued well beyond this threshold. A total of 1619 questionnaires were received and all were retained for analysis.

Values that were biologically implausible were treated as missing at the level of the individual field rather than by excluding the whole record. Height outside 130 to 210 cm, weight outside 30 to 200 kg, body mass index outside 14 to 60 kg/m^2^, and age outside 18 to 95 years were set to missing. This affected 25 anthropometric entries and 2 age entries. Fourteen participants left the WHOQOL-BREF entirely blank and therefore contributed to prevalence estimates but not to quality of life analyses. Regression models were fitted on complete cases, giving 1593 observations for the logistic model and 1579 for the linear models.

### 2.3. Data Collection Tool

The questionnaire was self-administered in Arabic and opened with a detailed explanation of the study aims, participant rights and instructions for completion. It comprised four sections: (A) informed consent; (B) sociodemographic and health-related characteristics; (C) dermatological disease information; and (D) the Arabic version of the WHOQOL-BREF. Responses were monitored throughout the collection period.

### 2.4. Measurements

#### 2.4.1. Sociodemographic and Anthropometric Variables

Participants reported nationality, sex, age, place of residence, educational level, monthly household income and marital status. Weight and height were self-reported and body mass index was calculated as weight in kilograms divided by height in metres squared.

#### 2.4.2. Psoriasis Status and Clinical Features

Psoriasis was ascertained by a single question asking whether the participant had been diagnosed with psoriasis, accompanied by a lay description of the condition as a chronic immune-mediated skin disease producing red patches covered by white or silvery scale, sometimes with itching. All participants answering affirmatively were classified as having psoriasis, irrespective of who made the diagnosis. Those answering affirmatively then reported the healthcare provider responsible for the diagnosis, age at first diagnosis, first-degree family history, symptoms experienced, presence of joint involvement, current symptom activity, number of flares during the preceding year, body areas affected, aggravating factors and treatments ever used.

#### 2.4.3. Lifestyle and Comorbidity Variables

Smoking status was recorded as never, current or ex-smoker. Physical activity was categorised as none during the week, moderate or vigorous activity for less than 30 min on five days per week, or moderate or vigorous activity for at least 30 min on five days per week. Participants also reported physician-diagnosed diabetes mellitus, hypertension, hyperlipidaemia, asthma and allergic rhinitis.

#### 2.4.4. Quality of Life Assessment

Quality of life was measured with the Arabic version of the WHOQOL-BREF, a 26-item generic instrument developed by the World Health Organization [18] and validated for use in Arabic-speaking populations [19]. Items 3, 4 and 26 were reverse-scored before analysis. Twenty-four items were assigned to four domains: physical health (7 items), psychological health (6 items), social relationships (3 items) and environment (8 items). The two remaining standalone items, covering the participant’s overall rating of quality of life and satisfaction with health, form the overall quality of life and general health facet and were analysed separately as recommended by the instrument developers [18]. Raw domain scores were transformed to a 0 to 100 scale, with higher scores indicating better quality of life [20].

### 2.5. Statistical Analysis

Data were screened in Microsoft Excel and analysed in R (version 4.2.3; R Foundation for Statistical Computing, Vienna, Austria). Continuous variables were summarised as means with standard deviations, and medians with interquartile ranges where distributions were skewed. Categorical variables were expressed as frequencies and percentages. The prevalence of psoriasis, and proportions within small subgroups of affected participants, were reported with 95% Wilson confidence intervals.

Continuous variables were compared between participants with and without psoriasis using the Mann–Whitney U test, since age and the quality of life scores departed from normality. Categorical variables were compared using the chi-square test, or Fisher’s exact test where any expected cell count fell below five.

A multivariable logistic regression model was fitted to identify factors independently associated with psoriasis, with adjusted odds ratios and 95% confidence intervals reported and model fit expressed as Tjur’s R2. Five separate multiple linear regression models were fitted for the overall quality of life facet and for each of the four domains, with results reported as unstandardised coefficients (β) and 95% confidence intervals. The logistic model was adjusted for age, sex, education, income, marital status, smoking status, physical activity, body mass index, urban or rural residence and hypertension. The quality of life models were adjusted for psoriasis status, age, sex, nationality, education, income, marital status, smoking status, physical activity, body mass index, diabetes and hypertension. Predictors were retained on conceptual grounds rather than selected by significance, and multicollinearity was checked using variance inflation factors, none of which exceeded conventional thresholds. All tests were two-tailed with significance set at α = 0.05. The study is reported in accordance with the STROBE guidelines.

The complete-case logistic model included 16 parameters for 52 events, approximately three events per variable, which is below the conventional threshold of ten [21]. Two sensitivity analyses were therefore performed. The full model was refitted using Firth’s penalized likelihood, which reduces small-sample bias in maximum likelihood estimates [22], with profile penalized likelihood confidence intervals [23]. A parsimonious model restricted to a priori covariates (age, sex, smoking status and body mass index) was also fitted, giving approximately ten events per variable. The prevalence estimate and the logistic models were then repeated after restricting cases to the 44 participants whose diagnosis had been made by a physician.

### 2.6. Ethical Considerations

The study protocol was approved by the Standing Committee for Scientific Research Ethics at Jazan University (Reference No. REC-47/03/1580, dated 4 September 2025). All procedures were conducted in accordance with the Declaration of Helsinki (1964) and its later amendments. Participants received an explanation of the study aims and their rights before taking part, and electronic informed consent was obtained from every respondent. Participation was voluntary and anonymous, no identifying information was collected, and responses were stored securely and accessible only to the research team.

### 2.7. Use of Generative Artificial Intelligence

Generative AI was used solely for language editing. Study design, data, analysis, and interpretation were entirely conducted by the authors.

## 3. Results

### 3.1. Sample Characteristics

A total of 1619 adults completed the questionnaire. Participants were predominantly female (1120, 69.2%) and Saudi nationals (1522, 94.0%), with a mean age of 27.5 ± 11.4 years (median 23, IQR 20 to 31). Most lived in urban areas (1207, 74.6%) and held a university degree (1113, 68.7%). Seventy percent were single (1134), and one-third reported a monthly household income below 5000 SAR (540, 33.4%) (Table 1). All five administrative regions were represented, with 940 participants (58.1%) from the Southern region, 418 (25.8%) from the Western region, 131 (8.1%) from the Eastern region, 101 (6.2%) from the Central region and 29 (1.8%) from the Northern region.

### 3.2. Health, Habitual and Anthropometric Characteristics

Mean body mass index was 23.6 ± 5.3 kg/m^2^, with a mean weight of 61.7 ± 16.9 kg and a mean height of 161.5 ± 9.1 cm. Never-smokers made up 85.2% of the sample (1380), while 155 participants (9.6%) were current smokers and 84 (5.2%) were ex-smokers. Regular physical activity of at least 30 min on five days per week was reported by 693 participants (42.8%), whereas 589 (36.4%) reported no weekly activity.

Chronic conditions were uncommon. Allergic rhinitis was the most frequent (285, 17.6%), followed by hyperlipidaemia (136, 8.4%), asthma (122, 7.5%), hypertension (96, 5.9%) and diabetes mellitus (91, 5.6%) (Table 2).

### 3.3. Prevalence and Clinical Characteristics of Psoriasis

**Figure 1:**
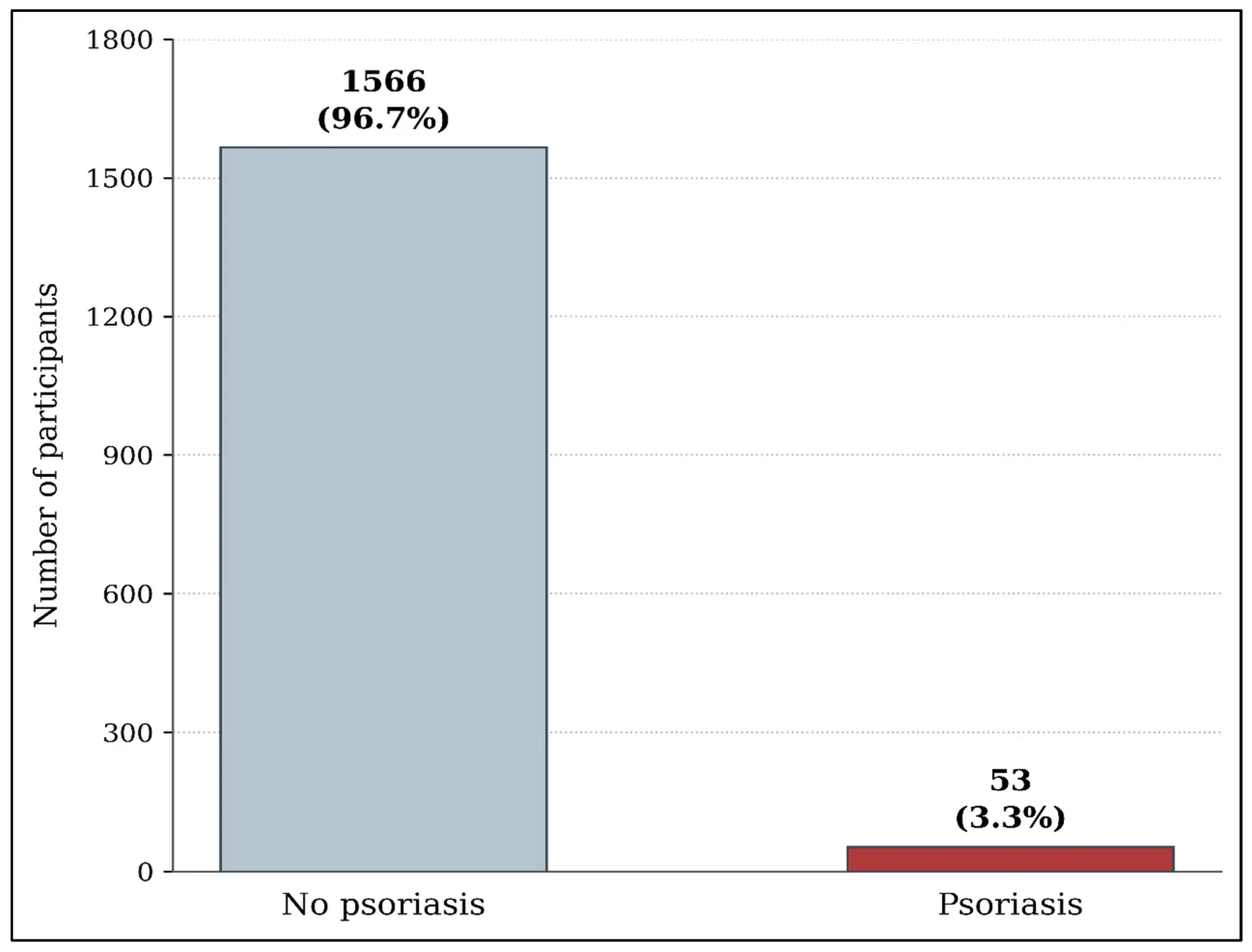
Prevalence of self-reported psoriasis in the study sample (N = 1619). Values are frequencies with corresponding percentages.

Fifty-three participants reported a diagnosis of psoriasis, giving a prevalence of 3.3% (95% CI 2.5 to 4.3) (Figure 1). The diagnosis had been made by a dermatologist in 30 cases (57.7%) and by a general practitioner in 13 (25.0%), while 8 participants (15.4%) described it as self-made or uncertain and 1 was diagnosed by another specialist. Restricting the case definition to the 44 participants diagnosed by a physician gave a prevalence of 2.7% (95% CI 2.0 to 3.6.

The mean age at first diagnosis was 19.8 ± 11.5 years (median 18, IQR 13 to 22), and 44 of 52 participants with available data (84.6%) were diagnosed before the age of 35. A first-degree family history was reported by 22 participants (42.3%), and 11 of 47 (23.4%) reported associated joint involvement. Symptoms were active at the time of the survey in 35 participants (67.3%). Over the preceding year, 21 (44.7%) reported fewer than five flares and 16 (34.0%) reported five or more, while 10 (21.3%) reported none.

Itching was the most frequently reported symptom (47, 90.4%), followed by dryness and scaling (43 each, 82.7%), redness (41, 78.8%) and skin discolouration (32, 61.5%). The legs were the most commonly affected site (28, 53.8%), followed by the scalp and arms (25 each, 48.1%), the hands (23, 44.2%) and the face (11, 21.2%). Psychological stress was the most frequently identified trigger (36, 69.2%), ahead of skin products such as perfumes and soaps (29, 55.8%), cold weather (22, 42.3%) and sun exposure (20, 38.5%). Topical preparations were the most widely used treatment (46, 88.5%). Herbal or alternative remedies had been tried by 18 participants (34.6%), exceeding the use of oral systemic agents (13, 25.0%), phototherapy (8, 15.4%) and biologic injections (4, 7.7%) (Table 3).

Considered together, only 20 of 52 participants with treatment data (38.5%, 95% CI 26.5 to 52.0) had ever received any systemic agent or phototherapy, while 32 (61.5%, 95% CI 48.0 to 73.5) had received neither. This gap persisted among those with the most active disease. Of the 16 participants reporting five or more flares in the preceding year, 11 (68.8%, 95% CI 44.4 to 85.8) had never received systemic therapy or phototherapy, as had 21 of the 35 participants (60.0%, 95% CI 43.6 to 74.4) who were symptomatic at the time of the survey. Among the 11 participants reporting associated joint symptoms, 9 (81.8%, 95% CI 52.3 to 94.9) had never received systemic therapy.

### 3.4. Factors Associated with Psoriasis in Bivariate Analysis

Psoriasis prevalence differed significantly across four characteristics. It was more than twice as high among current smokers as among never-smokers (7.7% vs. 2.8%, *p* = 0.004). Prevalence rose with education (4.0% among university-educated participants vs. 1.4% among those with high school education or lower, *p* = 0.029) and peaked in the middle-income bracket (6.2% in the 10,000 to 14,999 SAR group vs. 2.0% in the lowest, *p* = 0.012). It was also higher among participants reporting at least 30 min of activity on five days per week than among the inactive (4.6% vs. 2.4%, *p* = 0.031). Participants with psoriasis were older than those without (29.1 ± 10.2 vs. 27.4 ± 11.5 years, *p* = 0.014).

Body mass index did not differ between the groups (23.9 ± 4.4 vs. 23.6 ± 5.4 kg/m^2^, *p* = 0.327), and neither sex, nationality, residence, marital status nor any of the five chronic conditions examined were associated with psoriasis.

Participants with psoriasis scored lower on every WHOQOL-BREF measure. The difference reached statistical significance on the overall quality of life and general health facet (67.6 ± 24.1 vs. 74.6 ± 21.2, *p* = 0.037). At item level, 56.6% of participants with psoriasis rated their quality of life as good or very good, compared with 76.9% of those without (*p* = 0.001), and 58.5% were satisfied with their health, compared with 72.6% (*p* = 0.036). Differences across the four domains were smaller and none reached significance, with the environment domain coming closest (59.6 ± 22.1 vs. 65.7 ± 21.2, *p* = 0.054), although this borderline value should be interpreted with caution because no correction for multiple testing was applied (Table 4).

### 3.5. Factors Independently Associated with Psoriasis

In the multivariable logistic regression model, current smoking showed the largest adjusted association with psoriasis. Current smokers had more than four times the odds of reporting psoriasis compared with never-smokers (aOR 4.23, 95% CI 1.97 to 9.08, *p* < 0.001), while former smokers did not differ significantly (aOR 1.79, 95% CI 0.49 to 6.54, *p* = 0.377).

Two socioeconomic factors remained independently associated with psoriasis. Participants with a university education had higher odds than those with high school education or lower (aOR 2.56, 95% CI 1.07 to 6.13, *p* = 0.035), as did those in the 10,000 to 14,999 SAR income bracket relative to the lowest bracket (aOR 2.75, 95% CI 1.23 to 6.16, *p* = 0.014). The highest income bracket showed no such elevation (aOR 1.01, 95% CI 0.41 to 2.47, *p* = 0.989).

Male sex was associated with lower odds of psoriasis (aOR 0.41, 95% CI 0.19 to 0.90, *p* = 0.025). Age, body mass index, marital status, urban or rural residence, physical activity and hypertension were not independently associated with psoriasis. The model explained 9.0% of the variance in psoriasis status (Tjur’s R2 = 0.090) (Table 5).

The pattern was similar in sensitivity analyses. With Firth’s penalized likelihood, current smoking (aOR 4.17, 95% CI 1.92 to 8.62, *p* < 0.001), male sex (aOR 0.43, 95% CI 0.19 to 0.89, *p* = 0.023), university education (aOR 2.34, 95% CI 1.08 to 5.96, *p* = 0.030) and the 10,000 to 14,999 SAR income bracket (aOR 2.63, 95% CI 1.21 to 5.90, *p* = 0.015) remained associated with psoriasis. In the parsimonious model, current smoking (aOR 3.82, 95% CI 1.85 to 7.90, *p* < 0.001) and male sex (aOR 0.43, 95% CI 0.21 to 0.89, *p* = 0.024) retained their associations. When cases were restricted to physician-diagnosed psoriasis, current smoking (aOR 5.61, 95% CI 2.46 to 12.81, *p* < 0.001) and male sex (aOR 0.35, 95% CI 0.15 to 0.83, *p* = 0.017) persisted, the income association strengthened (10,000 to 14,999 SAR: aOR 4.33, 95% CI 1.71 to 11.01, *p* = 0.002), and the association with university education was no longer significant (aOR 2.15, 95% CI 0.88 to 5.24, *p* = 0.091).

### 3.6. Factors Associated with Quality of Life

Separate multiple linear regression models were fitted for the overall quality of life facet and for each of the four WHOQOL-BREF domains, adjusting for psoriasis status, sociodemographic characteristics, lifestyle factors and comorbidity.

Psoriasis was independently associated with a lower score on the overall quality of life and general health facet (β = −6.73, 95% CI −12.75 to −0.71, *p* = 0.028), a decrement of roughly one-third of a standard deviation on that measure. The coefficient for psoriasis was negative in all four domain models as well, but none reached statistical significance: social relationships (β = −6.04, 95% CI −12.82 to 0.73, *p* = 0.080), environment (β = −5.13, 95% CI −11.18 to 0.92, *p* = 0.097), physical health (β = −4.53, 95% CI −9.73 to 0.67, *p* = 0.088) and psychological health (β = −2.96, 95% CI −8.73 to 2.81, *p* = 0.314).

Household income was a significant predictor in all five models, with the strongest gradient in the environment domain, where the highest bracket scored 11.59 points above the lowest (95% CI 8.86 to 14.31, *p* < 0.001). Marital status was significant in four models, with married participants scoring consistently above single participants. Any physical activity was associated with higher scores than none in all five models. Hypertension was negatively associated with every outcome and produced the largest negative estimate in the social relationships domain (β = −10.07, 95% CI −15.59 to −4.55, *p* < 0.001). Diabetes was negatively associated with four of the five outcomes, and older age with three. Male sex predicted higher physical and psychological scores, while current smoking was associated only with the overall facet (β = −4.57, 95% CI −8.17 to −0.97, *p* = 0.013). Body mass index, nationality and education were not significant in any model (Table 6).

## 4. Discussion

In this cross-sectional study of 1619 adults residing in Saudi Arabia, psoriasis affected 3.3% of participants. Disease usually began early, with more than four in five cases diagnosed before 35, and psychological stress was the trigger most often identified. Current smoking showed the largest and most consistent adjusted association, with more than four times the odds of psoriasis relative to never-smoking. Participants with psoriasis rated their overall quality of life and general health significantly lower, although the four WHOQOL-BREF domains showed the same direction without reaching significance. To our knowledge this is the first study in Saudi Arabia to combine a community-based prevalence estimate, a clinical profile and a validated generic quality of life instrument in one sample.

### 4.1. Prevalence of Psoriasis

The prevalence of 3.3% sits at the upper end of the global range. Worldwide modelling places adult prevalence between roughly 0.1% and 3.0% [3], and Middle Eastern estimates have been flagged as uncertain because of sparse primary data [5]. Two considerations temper comparison. Ascertainment rested on self-reported diagnosis rather than examination, which inflates estimates relative to register-based studies, and an online convenience sample over-represents people with an interest in the topic. The estimate is therefore best read as sample-descriptive rather than nationally representative. Self-reported ascertainment could also inflate it, although the direction and size of any net bias cannot be determined from these data. Restricting cases to physician diagnoses lowered the estimate to 2.7%. For context, a population-based multinational survey that used random-digit dialling and did not require respondents to be under care reported a prevalence of psoriasis or psoriatic arthritis between 1.4% and 3.3% [24]. This limitation applies most directly to the prevalence estimate. Internal associations are in general less affected by selection than prevalence estimates, because bias arises mainly when entry into the sample depends jointly on the exposure and the outcome, but this cannot be verified in the present data, and the associations reported below should likewise be regarded as exploratory.

### 4.2. Clinical Profile and Treatment Patterns

The clinical picture matched established descriptions. Mean age at diagnosis was 19.8 years and 84.6% were diagnosed before 35, consistent with the early-onset peak of type I psoriasis [1]. A first-degree family history in 42.3% fits the strong hereditary component of early-onset disease [6]. Self-reported joint involvement in 23.4% is close to the pooled prevalence of 19.7% reported for psoriatic arthritis in a meta-analysis of 266 studies [25], though without rheumatological assessment this is symptom report rather than diagnosis. Psychological stress was named by 69.2%, exceeding all other triggers and echoing clinical series in which stress ranks first among patient-reported precipitants [6]. A meta-analysis found that almost half of patients regard their disease as stress-reactive and that stressful events preceding onset were associated with more than three-fold odds of psoriasis [26], although the direction and timing of this relationship remain debated [27].

The treatment pattern is the more striking finding. Topical preparations had been used by 88.5%, as expected, but herbal or alternative remedies had been tried by 34.6%, more than the 25.0% who had used oral systemic agents and far more than the 15.4% receiving phototherapy or the 7.7% receiving biologics. A clinic-based Saudi cohort reported comparably limited systemic therapy [11], and the northern-region study described a population managed largely with topicals [10]. Together these suggest that many people with psoriasis in Saudi Arabia may receive limited ongoing specialist management. Because disease severity, treatment indication and contraindications were not assessed, these figures describe the treatment received rather than the treatment required, and they cannot by themselves establish undertreatment. Limited use of systemic therapy has also been described elsewhere. In United States national surveys, up to 30% of patients with severe psoriasis received no treatment and, among those treated, more than a fifth received topical therapy alone [28], and in a multinational population survey more than 80% of patients with more extensive psoriasis were receiving no treatment or topical treatment only [24].

These subgroup proportions rest on small denominators, and their wide confidence intervals mean they should be read as indicative rather than definitive. Fewer than two in five participants had ever received any systemic agent or phototherapy, and the shortfall was concentrated among those with the most active disease rather than the mildest. More than two-thirds of those reporting five or more flares in the preceding year, and four in five of those reporting joint symptoms, had never received systemic therapy. Psoriatic arthritis carries a risk of irreversible structural damage, and diagnostic delay has been linked to poorer radiographic and functional outcomes [29]. Joint involvement here was self-reported and not confirmed rheumatologically, so these participants cannot be assumed to have psoriatic arthritis; the observation suggests only that rheumatological assessment of patients reporting joint symptoms may be worth examining. A pattern of this kind is observable only outside the clinic setting, because clinic-based samples enumerate patients already engaged with dermatological services and cannot quantify those who are not by design.

### 4.3. Smoking as a Determinant

Current smoking showed the largest adjusted association in the multivariable model, with an adjusted odds ratio of 4.23 (95% CI 1.97 to 9.08) and prevalence of 7.7% among current smokers versus 2.8% among never-smokers. This is directionally consistent with international evidence but larger than the pooled estimate of roughly 1.8 from a systematic review and meta-analysis [7]. The wide interval and modest number of exposed cases mean the point estimate warrants caution, and the model had approximately three events per variable, below the level at which maximum likelihood estimates are considered stable [21]. The association was nonetheless similar with Firth’s penalized likelihood (aOR 4.17), in a parsimonious model with approximately ten events per variable (aOR 3.82), and when cases were restricted to physician diagnoses (aOR 5.61), which suggests that its direction is robust even though its magnitude is imprecise. Ex-smokers did not differ significantly from never-smokers (aOR 1.79, 95% CI 0.49 to 6.54), a gradient compatible with the reversibility suggested by cohort data, although this design cannot establish temporality.

Biological plausibility is established. Nicotine influences keratinocyte proliferation and dendritic cell function, and cigarette smoke increases cutaneous oxidative stress, both converging on the interleukin-17 signalling that drives psoriatic inflammation [6,7]. The implication is direct: smoking is modifiable and cessation support is something dermatology services can deliver.

### 4.4. Socioeconomic and Demographic Associations

University education (aOR 2.56) and a monthly income of 10,000 to 14,999 SAR (aOR 2.75) were both independently associated with reporting psoriasis. Neither is readily interpreted as causal. The income relationship was not monotonic, since the highest bracket showed no elevation (aOR 1.01), which is not consistent with a dose–response effect and may indicate differential ascertainment. Better-educated participants with middle incomes may be likelier to seek assessment, receive a formal diagnosis and recognise the condition by name. This explanation cannot be tested with the present data, but these coefficients should not be read as risk factors.

Male sex was associated with lower odds (aOR 0.41, 95% CI 0.19 to 0.90). International epidemiology reports no meaningful sex difference in psoriasis prevalence [3], so this may reflect a sample that was 69.2% female together with differences in health reporting between women and men rather than higher biological risk, although the present design cannot distinguish between these explanations.

### 4.5. Psoriasis and Quality of Life

Psoriasis was independently associated with a 6.73-point reduction in the overall quality of life and general health facet, roughly one-third of a standard deviation. At item level, 56.6% of participants with psoriasis rated their quality of life as good or very good against 76.9% of those without, consistent with the well-documented impact of the disease on patient-reported outcomes [8]. The size of this impairment is in keeping with evidence that psoriasis carries a burden comparable to other major medical diseases [12] and with evidence that patients’ own rating of severity predicts quality of life more strongly than clinician-rated measures [13].

The domains told a more muted story. Coefficients were negative throughout, from −2.96 for psychological health to −6.04 for social relationships, but none reached significance. Two explanations apply and are not mutually exclusive. The first is statistical: with 53 cases, the domain-level estimates were imprecise, and their confidence intervals are compatible with both a modest effect and no effect. The second is conceptual: the two standalone items ask directly how a person rates their life and health, whereas each domain averages six to eight items covering areas such as transport, finances and access to information that a skin disease is unlikely to touch, diluting a genuine but focal effect. Reviews of quality of life measurement in psoriasis have made a similar case for dermatology-specific instruments such as the Dermatology Life Quality Index [17] when the aim is to capture disease-attributable burden [8]. The psychological domain showed the smallest coefficient despite the high prevalence of depressive symptoms reported in psoriasis [14], which may reflect the generic wording of its items rather than an absence of psychological burden.

### 4.6. Factors Associated with Quality of Life in the Wider Sample

Several factors unrelated to psoriasis showed stronger and more consistent associations with quality of life than psoriasis itself. Hypertension was negative in all five models and produced the largest single negative estimate observed, −10.07 points for social relationships. Diabetes was negative in four of five. Both conditions are more common among people with psoriasis [30], so cardiometabolic comorbidity may compound the burden of the skin disease. Income showed a graded positive relationship across every model, steepest in the environment domain where the highest bracket scored 11.59 points above the lowest, unsurprising given that this domain covers financial resources, housing and services. Married participants scored consistently above single participants, and any physical activity was associated with higher scores than none. An Italian cross-sectional study similarly found socioeconomic and psychosocial variables outweighing disease-specific measures in explaining quality of life among psoriasis patients [9]. These findings place the psoriasis effect in proportion: for an individual patient, comorbid cardiometabolic disease and social circumstance may matter as much as the skin condition.

### 4.7. Implications

Three tentative implications follow. Smoking cessation support may be worth considering as a routine component of psoriasis care, given the consistency of the association across model specifications and the modifiability of the exposure, although a cross-sectional design cannot establish that smoking precedes disease onset. The low uptake of phototherapy and biologics alongside substantial use of herbal remedies may indicate a gap between available treatment and the treatment patients receive, although this cannot be established without data on severity and treatment indication, and referral pathways and public information would be worth examining. Because stress was the commonest reported trigger and self-rated health was measurably lower among affected participants, psychological support deserves consideration within routine dermatological review [15]. These priorities align with the emphasis on prevention and integrated chronic disease management in Saudi Vision 2030.

### 4.8. Strengths and Limitations

The main strengths are the sample size, the community-based sampling frame, and use of a validated instrument scored per the developers’ protocol, including correct reverse-scoring and separate reporting of the overall facet. Recruiting outside dermatology services captured individuals who had never reached specialist care, a group invisible to earlier local studies. Participants were drawn from all five administrative regions, which to our knowledge has not previously been achieved in a Saudi community sample of this disease.

Several limitations apply. The cross-sectional design precludes causal inference, particularly for smoking, since behaviour after diagnosis cannot be distinguished from exposure preceding it. Psoriasis status was self-reported and unconfirmed by examination; 15.4% described their diagnosis as self-made or uncertain, and although all reported cases were analysed together, misclassification cannot be excluded and these participants reported the lowest quality of life scores in the sample. Convenience sampling over-represented women, younger adults, urban residents and the southern region, and anthropometric measures were self-reported. The prevalence estimate should accordingly be read as sample-descriptive rather than nationally representative. The adjusted associations may be less affected by the sampling design, although this cannot be verified in the present data, as noted in Section 4.1. Disease severity was not assessed objectively, so severity-related variation in quality of life could not be examined. With 53 cases, the domain-level estimates were imprecise, and the models explained a modest share of variance (Tjur’s R2 = 0.090; R2 between 0.040 and 0.095), indicating that much variation lies in unmeasured factors. The logistic model had approximately three events per variable; although Firth-penalized and parsimonious models gave consistent results, the adjusted odds ratios remain imprecise. Treatment-gap proportions within subgroups rest on small denominators. No correction for multiple testing was applied across the six regression models, so isolated *p*-values close to 0.05 should be interpreted with caution. Disease severity, treatment indication and contraindications were not recorded, so the treatment figures cannot be interpreted as undertreatment, and self-reported joint involvement cannot be equated with psoriatic arthritis. The study is therefore best regarded as an exploratory survey of self-reported psoriasis in an online convenience sample, and its estimates require confirmation in a probability-based sample with clinical verification of cases.

Future work should pursue three directions. A probability-based national sample with dermatologist verification would give a more reliable prevalence estimate and resolve the ascertainment questions raised here. A longitudinal design following smokers and never-smokers would test the temporal relationship with onset directly. Studies pairing a dermatology-specific instrument such as the Dermatology Life Quality Index with an objective severity measure would clarify how much of the burden is attributable to severity rather than to living with a visible chronic condition.

## 5. Conclusions

Psoriasis was reported by 3.3% of participants in this sample, began before the age of 35 in most cases, and was independently associated with poorer self-rated quality of life and general health. Current smoking was the most consistent modifiable factor associated with psoriasis across model specifications, while associations with education and income most likely reflect differences in diagnostic ascertainment rather than differences in risk. Herbal remedies had been used more often than systemic therapy and psychological stress was the most commonly reported trigger, patterns that may indicate gaps in access to evidence-based treatment and psychological support, although severity and treatment indication were not assessed and this cannot be established here. As an exploratory survey of an online convenience sample, the study identifies associations that require confirmation in a probability-based sample with clinically verified cases before they can guide practice.

## Figures and Tables

**Table 1:** Sociodemographic characteristics of participants (N = 1619).

Characteristic	*n*	%
**Age, years (mean ± SD)**	27.5 ± 11.4	
Median (IQR)	23 (20 to 31)	
**Sex**		
Female	1120	69.2
Male	499	30.8
**Nationality**		
Saudi	1522	94.0
Non-Saudi	97	6.0
**Residence**		
Urban	1207	74.6
Rural	412	25.4
**Education**		
High school or lower	437	27.0
University	1113	68.7
Postgraduate	69	4.3
**Monthly household income**		
<5000 SAR	540	33.4
5000 to 9999 SAR	367	22.7
10,000 to 14,999 SAR	276	17.0
≥15,000 SAR	436	26.9
**Marital status**		
Single	1134	70.0
Married	431	26.6
Divorced or widowed	54	3.3

**Table 2:** Anthropometric, behavioural and clinical characteristics of participants (N = 1619).

Characteristic	*n*	%
**Anthropometry (mean ± SD)**		
Weight, kg	61.7 ± 16.9	
Height, cm	161.5 ± 9.1	
Body mass index, kg/m^2^	23.6 ± 5.3	
**Smoking status**		
Never	1380	85.2
Current	155	9.6
Ex-smoker	84	5.2
**Physical activity**		
None during the week	589	36.4
<30 min, 5 days/week	337	20.8
≥30 min, 5 days/week	693	42.8
**Chronic conditions**		
Allergic rhinitis	285	17.6
Hyperlipidaemia	136	8.4
Asthma	122	7.5
Hypertension	96	5.9
Diabetes mellitus	91	5.6

**Table 3:** Prevalence of psoriasis and clinical profile of affected participants (*n* = 53).

Characteristic	*n*/Total	%
**Prevalence of psoriasis**	53/1619	3.3
**Diagnosed by**		
Dermatologist	30/52	57.7
General practitioner	13/52	25.0
Self-reported or uncertain	8/52	15.4
Other specialist	1/52	1.9
**Age at diagnosis, years (mean ± SD)**	19.8 ± 11.5	
Onset before age 35	44/52	84.6
**First-degree family history**	22/52	42.3
**Associated joint involvement**	11/47	23.4
**Currently symptomatic**	35/52	67.3
**Flares in the past year**		
None	10/47	21.3
Fewer than 5	21/47	44.7
5 or more	16/47	34.0
**Symptoms reported**		
Itching	47/52	90.4
Dryness	43/52	82.7
Scaling	43/52	82.7
Redness	41/52	78.8
Skin discolouration	32/52	61.5
**Sites affected**		
Legs	28/52	53.8
Scalp	25/52	48.1
Arms	25/52	48.1
Hands	23/52	44.2
Face	11/52	21.2
**Reported triggers**		
Psychological stress	36/52	69.2
Skin products	29/52	55.8
Cold weather	22/52	42.3
Sun exposure	20/52	38.5
**Treatments ever used**		
Topical preparations	46/52	88.5
Herbal or alternative remedies	18/52	34.6
Oral systemic agents	13/52	25.0
Phototherapy	8/52	15.4
Biologic injections	4/52	7.7

**Table 4:** Bivariate associations between participant characteristics, quality of life and psoriasis status.

Variable	Psoriasis (*n* = 53)	No Psoriasis (*n* = 1566)	*p*-Value
**Age, years (mean ± SD)**	29.1 ± 10.2	27.4 ± 11.5	0.014 *
**BMI, kg/m^2^ (mean ± SD)**	23.9 ± 4.4	23.6 ± 5.4	0.327
**Sex, *n* (%)**			0.246
Female	41 (3.7)	1079 (96.3)	
Male	12 (2.4)	487 (97.6)	
**Nationality, *n* (%)**			1.000
Saudi	50 (3.3)	1472 (96.7)	
Non-Saudi	3 (3.1)	94 (96.9)	
**Residence, *n* (%)**			0.338
Urban	43 (3.6)	1164 (96.4)	
Rural	10 (2.4)	402 (97.6)	
**Education, *n* (%)**			0.029 *
High school or lower	6 (1.4)	431 (98.6)	
University	45 (4.0)	1068 (96.0)	
Postgraduate	2 (2.9)	67 (97.1)	
**Income, *n* (%)**			0.012 *
<5000 SAR	11 (2.0)	529 (98.0)	
5000 to 9999 SAR	14 (3.8)	353 (96.2)	
10,000 to 14,999 SAR	17 (6.2)	259 (93.8)	
≥15,000 SAR	11 (2.5)	425 (97.5)	
**Marital status, *n* (%)**			0.088
Single	31 (2.7)	1103 (97.3)	
Married	21 (4.9)	410 (95.1)	
Divorced or widowed	1 (1.9)	53 (98.1)	
**Smoking status, *n* (%)**			0.004 **
Never	38 (2.8)	1342 (97.2)	
Current	12 (7.7)	143 (92.3)	
Ex-smoker	3 (3.6)	81 (96.4)	
**Physical activity, *n* (%)**			0.031 *
None	14 (2.4)	575 (97.6)	
<30 min, 5 days/week	7 (2.1)	330 (97.9)	
≥30 min, 5 days/week	32 (4.6)	661 (95.4)	
**Chronic conditions, *n* (%)**			
Allergic rhinitis	9 (3.2)	276 (96.8)	1.000
Hyperlipidaemia	6 (4.4)	130 (95.6)	0.445
Asthma	5 (4.1)	117 (95.9)	0.592
Hypertension	6 (6.2)	90 (93.8)	0.126
Diabetes mellitus	3 (3.3)	88 (96.7)	1.000
**WHOQOL-BREF (mean ± SD)**			
Overall QoL and general health	67.6 ± 24.1	74.6 ± 21.2	0.037 *
Physical health	62.9 ± 20.5	66.9 ± 18.1	0.357
Psychological health	60.2 ± 21.1	64.0 ± 19.8	0.276
Social relationships	60.5 ± 24.4	65.7 ± 23.2	0.160
Environment	59.6 ± 22.1	65.7 ± 21.2	0.054

Statistical significance: * *p* < 0.05; ** *p* < 0.01.

**Table 5:** Multivariable logistic regression model of factors independently associated with psoriasis.

Predictor	aOR	95% CI	*p*-Value
**Age (per year)**	1.01	0.97 to 1.05	0.767
**BMI (per kg/m^2^)**	1.00	0.93 to 1.06	0.878
**Male sex**	0.41	0.19 to 0.90	0.025 *
**Education (ref. high school or lower)**			
University	2.56	1.07 to 6.13	0.035 *
Postgraduate	1.49	0.27 to 8.22	0.646
**Income (ref. < 5000 SAR)**			
5000 to 9999 SAR	1.79	0.79 to 4.05	0.162
10,000 to 14,999 SAR	2.75	1.23 to 6.16	0.014 *
≥15,000 SAR	1.01	0.41 to 2.47	0.989
**Marital status (ref. single)**			
Married	1.09	0.46 to 2.60	0.847
Divorced or widowed	0.27	0.03 to 2.61	0.259
**Smoking (ref. never)**			
Current smoker	4.23	1.97 to 9.08	<0.001 ***
Ex-smoker	1.79	0.49 to 6.54	0.377
**Physical activity (ref. none)**			
<30 min, 5 days/week	0.87	0.34 to 2.23	0.777
≥30 min, 5 days/week	1.79	0.93 to 3.45	0.083
**Urban residence**	1.22	0.59 to 2.51	0.596
**Hypertension**	2.22	0.82 to 6.02	0.118
**Observations**	1593		
**Tjur’s R2**	0.090		

Statistical significance: * *p* < 0.05; *** *p* < 0.001.

**Table 6:** Factors significantly associated with WHOQOL-BREF scores.

Predictor	β	95% CI	*p*-Value
**Overall quality of life and general health facet (*n* = 1579, R2 = 0.095)**			
Psoriasis	−6.73	−12.75 to −0.71	0.028 *
Income 10,000–14,999 SAR (ref. < 5000)	5.39	2.31 to 8.46	<0.001 ***
Income ≥ 15,000 SAR (ref. < 5000)	5.87	3.15 to 8.58	<0.001 ***
Married (ref. single)	7.01	3.78 to 10.24	<0.001 ***
Divorced or widowed (ref. single)	10.75	4.31 to 17.19	0.001 **
Current smoker (ref. never)	−4.57	−8.17 to −0.97	0.013 *
Activity < 30 min (ref. none)	6.31	3.54 to 9.08	<0.001 ***
Activity ≥ 30 min (ref. none)	5.17	2.88 to 7.46	<0.001 ***
Diabetes mellitus	−11.58	−16.64 to −6.53	<0.001 ***
Hypertension	−11.22	−16.12 to −6.31	<0.001 ***
Age (per year)	−0.24	−0.38 to −0.10	<0.001 ***
**Physical health domain (*n* = 1579, R2 = 0.073)**			
Psoriasis	−4.53	−9.73 to 0.67	0.088
Male sex	4.45	2.39 to 6.51	<0.001 ***
Income 5000–9999 SAR (ref. < 5000)	3.47	1.11 to 5.83	0.004 **
Income 10,000–14,999 SAR (ref. < 5000)	5.90	3.24 to 8.56	<0.001 ***
Income ≥ 15,000 SAR (ref. < 5000)	6.51	4.17 to 8.86	<0.001 ***
Married (ref. single)	4.68	1.89 to 7.47	0.001 **
Divorced or widowed (ref. single)	7.40	1.83 to 12.97	0.009 **
Activity < 30 min (ref. none)	4.60	2.21 to 6.99	<0.001 ***
Activity ≥ 30 min (ref. none)	4.61	2.64 to 6.59	<0.001 ***
Diabetes mellitus	−4.52	−8.89 to −0.15	0.042 *
Hypertension	−7.21	−11.44 to −2.97	<0.001 ***
**Psychological health domain (*n* = 1579, R2 = 0.056)**			
Psoriasis	−2.96	−8.73 to 2.81	0.314
Male sex	4.06	1.77 to 6.35	<0.001 ***
Income 10,000–14,999 SAR (ref. < 5000)	5.17	2.22 to 8.12	<0.001 ***
Income ≥ 15,000 SAR (ref. < 5000)	5.81	3.21 to 8.40	<0.001 ***
Married (ref. single)	6.05	2.96 to 9.15	<0.001 ***
Divorced or widowed (ref. single)	11.02	4.85 to 17.19	<0.001 ***
Activity < 30 min (ref. none)	3.30	0.65 to 5.95	0.015 *
Activity ≥ 30 min (ref. none)	2.62	0.43 to 4.82	0.019 *
Diabetes mellitus	−5.83	−10.67 to −0.99	0.018 *
Hypertension	−5.82	−10.52 to −1.13	0.015 *
**Social relationships domain (*n* = 1579, R2 = 0.040)**			
Psoriasis	−6.04	−12.82 to 0.73	0.080
Income 10,000–14,999 SAR (ref. < 5000)	6.17	2.71 to 9.63	<0.001 ***
Income ≥ 15,000 SAR (ref. < 5000)	4.68	1.63 to 7.74	0.003 **
Married (ref. single)	7.29	3.66 to 10.93	<0.001 ***
Activity < 30 min (ref. none)	4.04	0.93 to 7.15	0.011 *
Activity ≥ 30 min (ref. none)	2.61	0.03 to 5.18	0.047 *
Hypertension	−10.07	−15.59 to −4.55	<0.001 ***
Age (per year)	−0.18	−0.33 to −0.02	0.028 *
**Environment domain (*n* = 1579, R2 = 0.082)**			
Psoriasis	−5.13	−11.18 to 0.92	0.097
Income 5000–9999 SAR (ref. < 5000)	3.97	1.23 to 6.72	0.005 **
Income 10,000–14,999 SAR (ref. < 5000)	9.66	6.56 to 12.75	<0.001 ***
Income ≥ 15,000 SAR (ref. < 5000)	11.59	8.86 to 14.31	<0.001 ***
Activity < 30 min (ref. none)	4.61	1.83 to 7.39	0.001 **
Diabetes mellitus	−6.25	−11.33 to −1.18	0.016 *
Hypertension	−6.16	−11.09 to −1.24	0.014 *
Age (per year)	−0.23	−0.37 to −0.09	0.001 **

Statistical significance: * *p* < 0.05; ** *p* < 0.01; *** *p* < 0.001.

## Data Availability

The dataset analysed during the current study is available from the corresponding author on reasonable request. The data are not publicly available because they contain responses collected under a confidentiality assurance to participants.

## Abbreviations

The following abbreviations are used in this manuscript:

aOR	Adjusted Odds Ratio
BMI	Body Mass Index
CI	Confidence Interval
WHOQOL-BREF	World Health Organization Quality of Life Brief Version
SAR	Saudi Arabian Riyal
SD	Standard Deviation
STROBE	Strengthening the Reporting of Observational Studies in Epidemiology
